# Prevalence of diabetic retinopathy among diabetic patients in Northwest Ethiopia—A cross sectional hospital based study

**DOI:** 10.1371/journal.pone.0262664

**Published:** 2022-01-21

**Authors:** Getasew Alemu Mersha, Yezinash Addis Alimaw, Asamere Tsegaw Woredekal

**Affiliations:** 1 Department of Optometry, School of Medicine, University of Gondar, Comprehensive Specialized Hospital, Gondar, Ethiopia; 2 Department of Ophthalmology, School of Medicine, University of Gondar, Comprehensive Specialized Hospital, Gondar, Ethiopia; Debre Tabor University, ETHIOPIA

## Abstract

**Background:**

Diabetic retinopathy is the most common microvascular complication of diabetes mellitus on eye and it is the leading cause of visual impairment among productive segment of the population. Globally, the prevalence of diabetic retinopathy is reported to be 27%. In Ethiopia, sufficient data is lacking on the prevalence of diabetic retinopathy as well as information on its predisposing factors. The study was required to assess the prevalence of diabetic retinopathy and its predisposing factors in diabetic patients attending at a General Hospital in Ethiopia.

**Methods:**

An institution based cross sectional study was employed on 331 diabetic patients recruited with a systematic random sampling technique. Data were collected through structured questionnaire, tracing patients’ medical folder and ocular health examination. Data were analyzed with Statistical Package for Social Science Version 20. Logistic regression methods of analysis were used to figure out predisposing factors of diabetic retinopathy. Adjusted odds ratio with 95% confidence interval was used to determine the strength of association.

**Result:**

A total of 331 diabetic patients completed the study with a response rate of 99.10%. The median duration of diabetes was 5 years. The prevalence of diabetic retinopathy was 34.1% (95%Confidence Interval (CI): 28.7%-39.3%). Low family monthly income (Adjusted Odds Ratio (AOR) = 7.43, 95% CI: 2.44–22.57), longer duration of diabetes (AOR = 1.44, 95% CI: 1.30–1.58), poor glycemic control (AOR = 4.76, 95%CI: 2.26–10.00), and being on insulin treatment alone (AOR = 3.85, 95%CI: 1.16–12.74) were independently associated with diabetic retinopathy.

**Conclusion and recommendation:**

The prevalence of diabetic retinopathy was 34.1%, higher than national and global figures. Low family monthly income, longer duration of diabetes, poor glucose control and being on insulin treatment alone were important risk factors of diabetic retinopathy. Proper diabetes self management and early screening of diabetic retinopathy in all diabetic patients were recommended.

## Introduction

Diabetes mellitus (DM) is a group of chronic metabolic conditions, all of which are characterized by elevated blood glucose levels resulting from the body’s inability to produce insulin or resistance to insulin action or both [1]. Worldwide, there are around 451 million diabetic patients [2] and the prevalence of diabetes mellitus in Ethiopia is 6.5%. The number of diabetic patient is expected to rise in the in the coming years due to rapid socio-demographic and economic transitions [3].

Persistently high glucose level in the blood causes generalized vascular damage resulting in various macro and micro vascular complications. Diabetic Retinopathy (DR) is a long term microvascular complication of diabetes on the eye. Without early detection and treatment, DR progress from its milder abnormalities to its advanced stages. Diabetic Retinopathy is complicated by macular edema, tractional retinal detachment and neovascular glaucoma which ultimately lead to a significant visual impairment [4].

Globally, the prevalence of DR in diabetic patients is estimated to be 27.0% [5]. Based on a pooled analysis of various hospital based studies the prevalence of DR is reported to be 31.6% in Africa [6] and 19.48%in Ethiopia [7]. According to the World Health Organization (WHO) it is estimated that DR makes up for37million cases of blindness in the world [8]. Visual loss from DR makes a challenging management of diabetic comorbidities and reduced life expectancy and diminish quality of life [9].

Longer duration of diabetes [10–16], high fasting blood sugar level [9, 15, 17, 18], presence of hypertension [15, 19, 20], obesity [21, 22], being on insulin treatment alone [12, 23–25]), presence of diabetes in a family [10, 18] and poor socio economic status [26, 27] were the most consistent factors associated with the development of DR in diabetic patients.

The risk of DR to sight can be reduced through good blood control, controlling of hypertension, effective early screening and having regular follow-up in a diabetic eye clinic. Diabetic retinopathy could be treated with timely laser treatment, intraocular injection of steroids, anti-vascular endothelial growth factor agents and intraocular surgery [28, 29]. The epidemiology and risk factors of DR has been adequately described in developed nations and a few numbers of studies have been attempted in the developing nations as well.

However, there is paucity of studies addressing the prevalence of DR and underlying risk factors in Ethiopia. Beside, no previous study was available in the study area before. Therefore, this study aimed to assess the prevalence and associated factors of DR in a diabetic patients attending at Debre Tabor General Hospital (DTGH) Northwest, Ethiopia.

## Methods and materials

### Study design and period

This is an institution based cross sectional study which was conducted from September 07/2020-November 06/2020at DTGH, situated in Debre Tabor town, the capital city of South Gondar Zone of the Amhara National Regional State, and it is located 667 km from Addis Ababa.

### Source and study population

In Ethiopia, diagnosis, management and supervision of diabetic patients have been done in the primary and secondary as well as tertiary health care facilities. They have been also followed with physicians and endocrinologists in diabetic clinic at general and tertiary hospitals. Patients would be linked to the eye care center only if they have developed other major complications or complained of any visual symptom. There is no a standardized screening program for DR as the patients first diagnosed with DM. This would significantly affect the prognosis of DR treatment, especially for patients who are coming from remote areas. Our study was targeted at all diabetic patients who were presented to the diabetic clinic at DTGH. This study included all types of diabetic patients who were aged 18 years and above at DTGH during the course of the study.

### Exclusion criteria

- Patient with pregnancy induced diabetes (gestational diabetes),
- Patients who were severely ill, uncomfortable for the interview
- Patients who were unable to be seated and examined with slit lamp indirect ophthalmoscope.

### Sample size calculation

**Fig 1:**
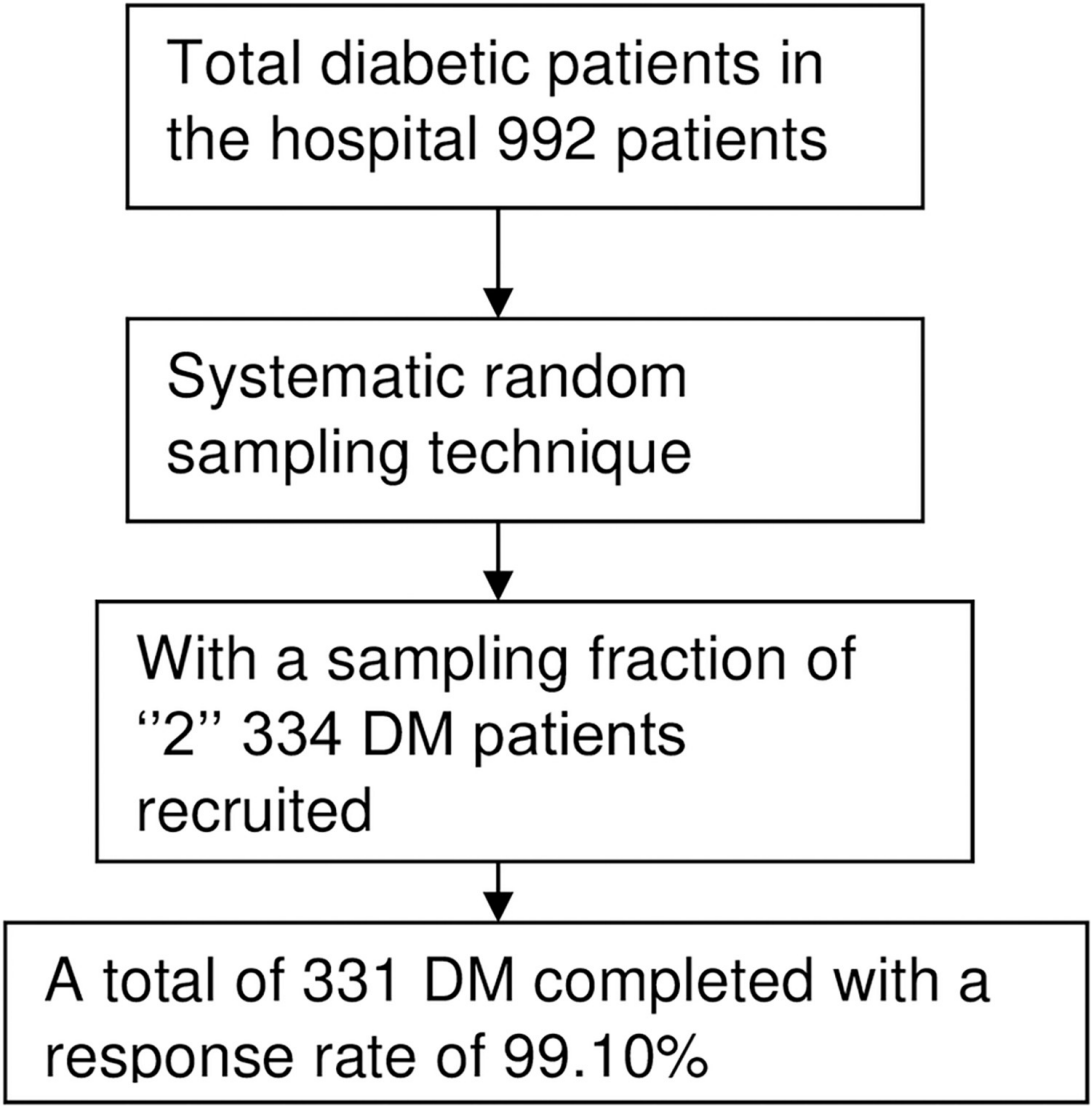
A flow chart showing the sampling and data collection procedure.

The required sample size was determined in advance based on a single population proportion formula by taking 25.5% prevalence figure from a similar study in Bahir Dar Felege Hiwot Referral Hospital [30], 95% CI, 4% margin of error, applying reduction formula to the size 992 total diabetes population at DTGH and 10% non-response rate. Accordingly, the final computed sample size was 344. Having a k value of 2, every other diabetic patient was approached for the study (Fig 1).

### Ethical consideration

Ethical approval was obtained from the University of Gondar, College of Medicine and Health Sciences, School of Medicine Ethical Review Board. Written informed consent was obtained from each study participants and confidentiality was kept through coding and locking the questionnaire. Patients of age below 21 were received the consent through their legal guardians or parents. The written informed consent was approved by the University of Gondar Ethical Review Board. Generally, the study was conducted intent of the Declaration of Helsinki.

### Operational definition

Glucose control was labeled as poor if a current FBS was >152 milligram/deciliter (American Diabetic Association Standards of Medical Care in Diabetes), the presence of hypertension was assured based on the diagnosis made by a physician and written in the patients’ medical folder. Ascertainment of DR was done based on the Early Treatment of Diabetic Retinopathy Study [31, 32].

### Data collection procedures and quality control

Interviewer-administered questionnaire, document review and ocular examinations were applied to collect the data. The questionnaire was adapted from reviewed literatures [15, 33–35] and has four sections; section I: contain questions on socio-demographic and economic variables, section II: Contain questions on behavioral variables, section III: contain questions related diabetic follow up and eye check up, section IV: checklist to trace data on clinical variables. The questioner was first prepared in English and translated to Amharic (local language) and back to English by independent interpreter to maintain consistency and clarity.

The Amharic version of the questionnaire was validated through pretesting on 5% of the sample and acronbach’s alpha value of 7.14 was obtained for these groups. Three trained BSc nurses and two optometrists were involved in the data collection process. The three BSc nurses interviewed the participants on socio-demographic (age, sex, educational status, occupational status, residence and family monthly income) Behavioral (alcohol consumption, physical activity and smoking status) and some of the clinical factors (family history of DM and duration of DM). They also traced data on the types of DM, medication used and presence of hypertension from the patient’s medical folder and measured height and weight of participants.

### Diagnosis of diabetic retinopathy

Experienced optometrists had performed the diagnosis of DR using a90 diopter of Volk lens and slit lamp biomicroscope after the eye was treated with 1% tropicamide eye drop. Each eye got examined by the two optometrists who were double masked for their findings. In case of conflict, a senior ophthalmologist was consulted to reach an agreed diagnosis of DR. Labeling of DR was made based on the protocol of Early Treatment Disease Retinopathy Study.

### Data processing and analysis

After the data was checked for completeness it was transcribed into EPI INFO 7 and exported to SPSS version 20. Data was cleaned, coded, merged and analyzed. The descriptive statistics was summarized and presented using frequency tables. Bivariable logistic regression method of analysis was used to identify candidate variables. Variables with p-value < 0.2 in bivariable logistic regression were entered into a multivariable logistic regression method of analysis. Variables having p-values < 0.05 were considered as statistically significant. The model fitness was ensured by Hosmer and Lemeshow’s goodness of fit test.

## Result

### Socio demographic characteristics of diabetic patients

A total of 331 diabetes patients with a mean age of 45.48 (SD±16.88) years were involved in the study. Most of the diabetic patients were male 181 (54.7%), married 220 (66.5%), had no formal education 188 (56.8%), were farmer 99 (29.9%) and urban residents 205 (63.1%). The median family monthly income of the respondents was 3000 Ethiopian Birr (ETB) (IQR = 4193Ethiopian Birr) (Table 1).

**Table 1:** Socio-demographic and economic characteristics of study participants at Debre Tabor General Hospital, Northwest Ethiopia, 2020 (n = 331).

Variable	Frequency	Percent
**Gender**		
Male	181	54.7
Female	150	45.3
**Age (Years)**		
18–27	61	18.0
28–37	55	16.6
38–47	59	17.8
≥48	156	47.1
**Marital status**		
Single	111	33.5
Married	220	66.5
**Educational status**		
No formal education	188	56.8
Primary and secondary school	72	21.8
Collage and above	71	21.5
**Occupational status**		
Government employee	56	16.9
Retired	25	7.6
House wife	47	14.2
Farmer	99	29.9
Other	52	15.7
No job	52	15.7
**Residence**		
Urban	205	61.9
Rural	126	38.1
**Family monthly income (Ethiopian Birr)**		
≤ 2000	126	38.1
2001–3577	47	14.2
3578–6500 ≥6501	82 76	24.8 23.0

n = Sample Size.

### Clinical and behavioral characteristics of diabetic patients

The maximum duration of diabetes was 40 years and the minimum was 3 months since diagnosis. The median duration of diabetes was 5 (IQR = 8) years and the median level of FBS was 150 (IQR = 94)mg/dl. Majority of the diabetic patients were type I DM 183 (55.3%), had a good glycemic control 168 (50.8%), used insulin alone as a treatment modality 191 (57.7%), non drinkers 204 (61.6%) and had moderate level of physical activity 185 (55.9%) (Table 2).

**Table 2:** Clinical and behavioral characteristics of study participants at Debre Tabor General Hospital Northwest Ethiopia, 2020 (n = 331).

Variable	Frequency	Percent
**Body mass index**		
≤24.99	245	74.0
25–29.9	58	17.5
≥30	28	8.5
**Type of diabetes**		
Type I	183	55.3
Type II	148	44.7
**Duration of diabetes (in years)**		
<5	162	48.9
≥5	169	51.1
**Glycemic control**		
Good control	168	50.8
Poor control	163	49.2
**Mode of treatment**		
Insulin alone	191	57.7
Tablet	106	32.0
Combined	34	10.3
**Drinking status**		
Non drinker	204	61.6
Moderate drinker		
	102	30.8
Heavy drinker	25	7.6
**Physical activity**		
Physical inactivity	61	18.4
Low physical activity	85	25.7
Moderate physical activity	185	55.9

n = Sample Size.

### Systemic co morbidity and follow up of the diabetic patients

From the total participants, 102 (30.8%) had hypertension as a co-morbidity, 246 (74.3%) visited the DM clinic every month, 84 (25.4%) had family history of DM, 139 (42.0%) had no prior eye exam and only 87 (26.1%) had awareness about DR (Table 3).

**Table 3:** Systemic comorbidity, frequency of visit and awareness characteristics of study participants at Debre Tabor General Hospital, Northwest Ethiopia, 2020 (n = 331).

Variables	Frequency	Percent
**Hypertension**		
Yes	102	30.8
No	229	69.2
**Frequency of DM clinic visit**		
Every month	246	74.3
Every two months	85	25.7
**Family history of DM**		
Yes	84	25.4
No	247	74.6
**History of eye exam**		
Yes	192	58.0
No	139	42.0
**Awareness of DR**		
Yes	87	26.3
No	244	73.7

**n** = Sample Size.

### Prevalence of diabetic retinopathy among diabetic patients

From the total diabetic patients recruited in the study the prevalence of DR was 113 (34. 1%) (95%CI: 28.7%-39.3%). The prevalence figure was 34.4% among type 1 DM and 33.8% among type 2 DM.

### Related factors to diabetic retinopathy in diabetic patients

From bivariable logistic regression methods of analysis, age, occupational status, family monthly income, duration of diabetes, glycemic control, mode of treatment, hypertension, BMI, and family history of diabetes were found to be statistically and significantly associated with DR. Subsequently, on a multivariable logistic regression method of analysis family monthly income, duration of diabetes, glycemic control and mode of treatment were found to have a statistically significant association with DR (Table 4).

**Table 4:** Factors associated with diabetic retinopathy in diabetes patients at Debre Tabor General Hospital, Northwest Ethiopia 2020 (n = 331).

Variable	Diabetic Retinopathy n (%)				
	Yes No	COR (95%CI)		AOR (95%CI)	
**Age (Years)**					
18–27	10 (16.4%) 51 (83.6%)	1	1		
28–37	16 (29.1%) 39 (70.9%)	2.09 (0.81–5.38)	2.31 (0.65–8.21)		
38–47	23 (39.0%) 36 (61.0%)	3.26 (1.05–6.49)*	2.09(0.48–9.18)		
≥48	64 (41.0%) 92 (59.0%)	3.55 (1.68–7.50)*	1.90 (0.42–8.63)		
**Occupational status**					
Government employee	14 (25.0%) 42 (75.0%)	1	1		
Retired	14 (56.0%) 11 (44.0)	3.82 (1.41–10.32)*	6.55 (0.32–32.27)		
House wife	20 (42.6%) 27 (57.4%)	2.22 (0.96–5.13)	1.43 (0.27–7.64)		
Farmer	38 (38.4%) 61 (61.6%)	1.87 (0.90–3.87	1.76 (0.33–9.32)		
Other	13 (25.0% 39 (75.0%)	1.00 (0.42–2.39)	0.68 (0.12–3.70)		
No job	14 (26.9%) 38 (73.1%)	1.11 (0.47–2.62)	0.93 (0.17–4.96)		
**Family monthly income (ETB)**					
≤ 2000	58 (46.0%) 68 (54.0%)	5.04 (2.43–10.44)**	7.43 (2.44–22.57)**		
2001–3577	19 (40.4%) 28 (59.6%)	4.01 (1.69–9.52)*	3.65 (1.05–12.69)*		
3578–6500	25 (30.5% 57 (69.5%)	2.59 (1.17–5.73)*	4.58 (1.49–14.11)*		
≥6501	11 (14.5%) 65 (85.5%)	1	1		
**Duration of DM**		1.46 (1.34–1.58)**	1.44 (1.30–1.58)**		
**Glycemic control**					
Good control	36 (21.4%) 132 (78.6%)	1	1		
Poor control	77 (47.2%) 86 (52.8%)	3.28 (2.03–5.30)**	4.76 (2.26–10.0)**		
**Mode of treatment**					
Insulin alone	76 (39.8%) 115 (60.2%)	1.21 (0.57–2.59)	3.85 (1.16–12.74)*		
Tablet	25 (23.6%) 81 (76.4%)	0.57 (0.25–1.30)	0.84 (0.23–3.02)		
Combined	12 (35.3%) 22 (64.7%)	1	1		
**Body Mass Index**					
≤24.99	80 (32.7%) 165 (67.3%)	1	1		
25–29.9	18 (31.0%) 40 (69.0%)	0.93 (0.50–1.72)	1.14 (0.41–3.16)		
≥30	15 (53.6%) 13 (46.4%)	2.38 (1.08–5.24)*	1.37 (0.34–5.49)		
**Hypertension**					
Yes	45 (44.1%) 57 (55.9%)	1.87 (1.15–3.03)*	2.21 (0.88–5.56)		
No	68 (27.5%) 161 (70.3%)	1	1		
**Family history of DM**					
Yes	45 (53.6%) 39 (46.4%)	3.04 (1.82–5.07)**	1.82 (0.81–4.09)		
No	68 (27.5%) 179 (72.5%)	1	1		

**n** = sample size, **ETB** = Ethiopian Birr

*p value <0.05

** p-value<0.001. Hosmer and Lemeshow Test = **0.445.**

Regarding family monthly income of diabetic patients, those who had an income of ≤2000 ETB were 7.43 times (AOR = 7.43, 95% CI: 2.44–22.57), income from 2001–3577 ETB were 3.65 times (AOR = 3.65, 95% CI: 1.05–12.69) and income 3578–6500 were 4.58 (AOR = 4.58, CI: 1.49–14.11) more likely to develop DR compared to those who had income of >6501ETB. With respect to the duration of diabetes, there was a 1.44 times increase in the presence of DR for a one year up in the duration of diabetes (AOR = 1.44, 95% CI: 1.30–1.58). The risk of developing DR was nearly 5 times higher in diabetic patient who had a poor glycemic control (AOR = 4.76, 95%CI: 2.26–10.00), than those who had a good glycemic control.

Diabetic patients who were only under insulin treatment alone were nearly 4 times (AOR = 3.85, 95%CI: 1.16–12.74) more likely to have DR compared to those who were under combined treatment.

However, in this study factors such as, gender, marital status, educational status, residence, physical activity, alcohol drinking status, types of diabetes, body mass index, frequency of clinical visit in DM clinic, and having awareness about diabetic retinopathy did not show statistically significant association with DR either on a bivariable or a multivariable logistic regression analysis.

## Discussion

The present study has shown that, the prevalence of DR among diabetic patients was34.10%, comparable with previous hospital based studies in Nigeria (36%) [36] and Bangladesh (36.10%) [37]. The prevalence figure was higher than priorly reported from BahirDar (25.50%) [30], Gondar (17.00%) [38], Arba Minch (13.00%) [10], in Ethiopia however, it was lower compared to previous reports in Addis Ababa (50.1%) [39] and Jimma [15] (41.10%).

The higher prevalence rate of DR in the current study might be due to the utilization of dilated fundus examination technique which helps to ascertain more number of cases with DR, while the previous hospital based studies in Ethiopia (Bahir Dar, Gondar and Arba Minch) reported DR based on referring the patients’ medical chart. Hence, diabetic patients rarely check on their visual status in the absence of symptoms and complete recording of findings may not be available, labeling DR based on reviewing patients’ medical chart could result in a lower estimate of DR. The former studies in Addis and Jimma were at referral centers; included severely manifesting diabetic patients with multiple comorbidities and diabetic patients with relatively longer duration that could generally increase diabetic complications including DR.

On the other hand the prevalence figure was higher compared to previous hospital based studies in African Nations such as, Uganda (19%) [40], Zimbabwe (28.40%) [13], Tanzania (27.90%) [41], and Egypt (24.00%), [24]. However, it was lower in contrast to Sudan [20] (82.60%), Zambia [42] (52.00%) and Cameroon [43] (40.30%), The variation may result from the discrepancy in sample size, nature of the study population and methods of screening DR.

The prevalence figure was also higher compared to previous hospital based studies in Middle East and Asian countries like Saudi Arabia (16.0%), ([23] Pakistan (17.00%), [16] China (27.90%) [17], however it was exceptionally lower from India [18] (60.90%).

The discrepancy may result from the variation in sample size, inclusion and exclusion criteria. For instance, studies in Saudi Arabia and Pakistan were done relatively on small sample size of type 2 diabetic patients; excluding type 1 diabetic patient in these studies could underestimates the magnitude of DR. The increased prevalence of DR in India might be due the fact that, their study was conducted at the ophthalmology clinic where cases of DR can be treated and followed, and to estimate the prevalence in this setting would result high prevalence figure on DR.

The prevalence rate was also higher compared to studies in Europe and United states including: United Kingdom (28.5), [44] Spain (14.90%), [12] Slovakia (15.50%), [14] New Zealand (22.50%) [45] and United States of America (USA) (14.70%) [46]. Conversely, it was lower compared to Pittsburgh USA [11] (44.00%).

The disparity could be related to the divergence in study population characteristics. For example, studies in Slovakia, Spain, United Kingdom, and USA were on a general diabetic population, unlike the current study which was based on clinic based diabetic population. Ascertainment of DR in clinic diabetic patients would result in a high prevalence figure. The study population in Pittsburgh USA were inpatient diabetic patients that might have been challenged by systemic co-morbidities predispose them to diabetic complications including DR.

A one year up in the duration of diabetes increase the chance of developing of DR and this finding was in line with studies in Arba Minch, Jimma and around the world [10–16] which showed that, longer duration of DM is significantly associated with the development of DR. This association could be explained in a way that widening of retinal artery is occurred with increment duration of diabetes, which is a sub clinical marker of endothelial dysfunction that leads to DR [28].

The likely hood of having DR was higher among diabetic patients with poor glycemic control as compared to those with good glycemic control. This result was supported by studies in Jimma [15], China [17], Bangladesh [37] and India [18]. High glucose level in the endothelial cells of the retinal artery leads to impaired glucose up take and increase oxidative stress to these cells which leads to diabetic complications like DR [28].

The risk of having DR was higher for diabetic patients having average monthly income of ≤ 2000, 2001–3577 and 3578–6500 ETB than diabetic patients with an income of ≥6500. This result was in agreement with what has been found in Sudan [26] and India [27] which indicated that, low socioeconomic status was a risk factor for the occurrence of DR. Diabetic patients with low monthly income have deficit to cover medication and food related expenditures; this gives them a challenging diabetes self management, linked to poor glycemic control ultimately lead to diabetic complications like DR.

Furthermore, the presence of DR was higher among diabetic patients who were under only insulin (injection) therapy as compared to those who were under combined therapy to manage their disease. This was corroborated with the finding of studies in Egypt, Saudi Arabia, Japan and USA [12, 23–25] which indicated that, diabetic patients with insulin therapy alone had an increased risk of developing DR. It has been suggested that, combined therapy can achieve better and faster achievement of a good glycemic target which helps to prevent the long term complication of diabetes such as DR [47].

### Limitations

The data was obtained from a few numbers of diabetic patients therefore; the estimation may not reflect the actual prevalence of DR and figure out its important predictors. Hence the study was based on hospital patients it does not represent the prevalence of DR in a general diabetic population. Finally, the assessment of DR was done based on ophthalmic examination rather than fundus photograph and this might have actually under estimated the magnitude of DR.

## Conclusion

In conclusion, the prevalence of DR in a diabetic patients attending DTGH was higher compared to the global and national prevalence DR. Moreover, the prevalence figure was higher than reports of previously conducted hospital based studies in Ethiopia. Low monthly income, longer duration of diabetes, poor glucose control and being on Insulin treatment alone were found to be the most important risk factors associated with the development of DR in diabetes.

Therefore, it is better to embark a coordinated early screening for DR in the hospital. It is also equally important to draw more attentions and increase effort in provision of affordable and accessible health care service for diabetic patients, here by reduce devastating consequences of the disease. It is also equally important to equip the primary diabetic clinics with a fundus camera for an early and effective detection of DR.

## Supporting information

S1 Dataset (SAV)

S1 File (DOCX)

## Abbreviations

AOR: Adjusted Odds Ratio
BMI: Body Mass Index
CI: Confidence Index
DM: Diabetes Mellitus
DR: Diabetic Retinopathy
DTGH: Debre Tabor General Hospital
ETB: Ethiopian Birr
EPEINFO: Epidemiological Information
IQR: Interquartile Range
SD: Standard Deviation
SPSS: Statistical Package for Social Science
USA: United States of America
