# Supplementary material for: Prevalence of diabetic retinopathy among diabetic patients in Northwest Ethiopia—A cross sectional hospital based study
Source: PLoS One. 2022 Jan 21;17(1):e0262664. doi: 10.1371/journal.pone.0262664 (PMC8782290; doi:10.1371/journal.pone.0262664)
Supplement: S1 File — (DOCX) [file pone.0262664.s002.docx]

**English version of the questionnaire**

**General information**

Serial no.______________ Card No______Kebele______

Data collection date_______________________________

Name of data collector_____________________________

Signature _______________________________________

**Section I: Assessment of Socio-demographic and Economic variables**

| **S.No** | **Question** | **Response** | **Remark** |
| --- | --- | --- | --- |
| 01. | Sex | 1. Male 2. Female |  |
| 02. | How old are you? | ------------Years |  |
| 03. | What is the highest level of education you have completed? | 1. Unable to read and write 2. Read and write 3. Primary school 4. Secondary school 5. High school/preparatory school 6. College/University completed |  |
| 04. | Occupational status? | 1. Government employee 2. Retired 3. House wife 4. Daily laborer 5. Merchant 6. Farmer 7. No job 8. Others, specify________ |  |
| 05. | Residence? | 1. Urban 2. Rural |  |
| 06. | Average Family Monthly income? | ___________ETB |  |

**Section II: Behavioral Measurements**

| **S.No** | **Question** | **Response** | **Remark** |
| --- | --- | --- | --- |
| 01. | Do you ever smoke cigarettes? | 1. Yes 2. No | **If no skip to question 05** |
| 02. | For how long do you smoke cigarettes? | 1. Below 1 year 2. 1-5 year 3. >5 year |  |
| 03. | Do you smoke daily? | 1. Yes 2. No |  |
| 04. | How many cigarettes per day? | ---------Number |  |
| 05. | Do you consume an alcoholic drink during the last 3 months? | 1. Yes 2. No | **If not skip to Q 09** |
| 06. | What type of alcohol did you drink? | 1. Beer 2. Wine 3. Tella/Local beer 4. Areki 5. Others, specify____ |  |
| 07. | On average how many glasses/bottles do you drink per day? | _____bottles/glasses/birlie/Tassa |  |
| 08. | How frequent did you drink alcoholic drink per week? | ---------days |  |
| 09. | Does your work involve vigorous-intensity activity that causes large increases in breathing or heart rate like [carrying or lifting heavy loads, digging or construction work] during the last 6 months? | 1. Yes 2. No | **If no skip to Q 13** |
| 10. | In a typical week, on how many days do you do vigorous-intensity activities as part of your work? | _____days |  |
| 11. | How much time do you spend doing vigorous-intensity activities at work on a typical day? | _____Hours/minutes |  |
| 12. | Do you walk or use a bicycle (pedal cycle) to go to and from places in the last 6 months? | 1. Yes 2. No | **If no skip to Q14** |
| 13. | In a typical week, on how many days do you walk or bicycle to get to and from places? | _______ days |  |
| 14. | How much time do you spend walking or bicycling for travel on a typical day? | _____Hours/Minutes |  |

**Section III: Questions related to diabetic follow up and eye check up**

| **S.No** | **Questions** | **Response** | **Remarks** |
| --- | --- | --- | --- |
| 01. | How long have you been with diabetes? | ______________Years |  |
| 02. | Is there anyone with diabetes in your family? | 1. Yes 2. No 3. Do not know |  |
| 03. | How often do you visit the diabetes clinic? | 1. Every Month 2. Every 2 month 3. Every 3 Month 4. Every 6month 5. Every year 6. Others specify |  |
| 04. | Have you been examined your eye before? | 1. Yes 2. No |  |
| 05. | Have you ever heard of diabetic retinopathy? | 1. Yes 2. No |  |

**Section IV: Clinical Data**

| **S.No** | **Question** | **Response** | **Remark** |
| --- | --- | --- | --- |
| 01. | Type of DM | 1. Type 1 2. Type 2 | From card records |
| 02. | Level of current Fasting Blood Sugar | _____mg/dl | " |
| 03. | Mode of treatment | Tablets 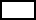  Injections 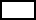  Diet 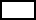  Both insulin and tablet 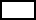 | " |
| 04. | Hypertension | 1.Yes 2.No | “ |
| 05. | Body Mass Index (BMI) | _____height(m) ____weight (kg) ___ | Through Measurements |
| 06. | Diabetic retinopathy | 1. Yes 2. No | Through ocular examination |

**Amharic version of questionnaire**

**የአማርኛመጠይቅቅጽ**

ጤናይስጥልኝእኔ ………………………………………እባላለሁ፡፡የመጣሁትከጎንደርዩኒቨርስቲነዉ፡፡በደብረታቦርጠቅላላሆስፒታልበስኳርሕመምታካሚዎችላይበስኳርህመምምክንያትየሚመጣየአይንየሁዋለኛዉየብርሐንመቀበያጉዳት/እክልእናተዛማጅምክንያቶችዙሪያየዳሰሳጥናትአባልነኝ፡፡እርስዎጥናቱንይሳተፉዘንድበአክብሮትእንጠይቃለን፡፡በስኳርህመምምክንያትየሚመጣየአይንየሁዋለኛዉየብርሐንመቀበያጉዳት/እክልእናተዛማጅምክንያቶችበተመለከተለምንጠይቅዎትጥያቄወችየሚሰጡንትክክለኛመልስእናዓይንዎትንበምንመረምርበትወቅትየሚያደርጉልንትብብርለጥናቱበጣምጠቃሚድርሻአለው፡፡ከእርስዎየሚወሰድማንኛውምመረጃበሚስጥርየተጠበቀሲሆንበጥናቱላይአሁንምሆነመሀልላይመሳተፍባይፈልጉተሳትፎውንማቋረጥመብትዎነዉ፡፡ነገርግንጥናቱከሚሰጠዉጥቅምአንጸርቢሳተፉደስይለናል፡፡መጠይቁንእናምርመራዉንለማጠናቀቅቢበዛ 25 ደቂቃይወስዳል፡፡ፍቃደኝነትዎንበንግግርዎእንዲያረጋግጡልንበትህትናእየጠየቅንወደቃለመጠይቁእናምርመራዉእንሄዳለን፡፡

ለመሳተፍፈቃደኛከሆኑወደሚቀጥለውገፅእለፍ.

ካልተስማሙምአመስግነህወደቀጣዩተሳታፊእለፍ

መረጃሰብሳቢ

ስም………………………………………………………….. ፊርማ………………………………. ቀን………………………..

ያረጋገጠዉተቆጣጣሪ

ስም……………………………………………………………. ፊርማ………………………….. ቀን…………………………

**አጠቃላይመረጃ**

01. መለያቁጥር------------------------- ቀበሌ -----------------

02. መጠይቁየተካሔደበትቀን --------------------/--------------/----------------

03. የመረጃሰብሳቢውስም-----------------------------------------------ፊርማ -----------------------

**ክፍልአንድ:-ማህበራዊናስነህዝባዊመረጃዎች**

| **ተ.ቁ** | **ጥያቄዎች** | **ምላሽ** | **ምርመራ** |
| --- | --- | --- | --- |
| 01. | ፃታ | 1. ወንድ 2. ሴት |  |
| 02. | እድሜዎስንትነው? | -----------ዓመት |  |
| 03. | የትምህርትደረጃ? | 1. ማንበብናመፃፍየማይችል/የማትችል 2. ማንበብናመፃፍየሚችል/የምትችል 3. የመጀመሪያት/ት (1-8) 4. 2ኛደረጃትምህርት (9-10) 5. መሰናዶትምህርት (11-12) 6. ኮሌጅ/ዩኒቨርሲቲያጠናቀቀ |  |
| 04. | የስራሁኔታ? | 1. የመንግስትሰራተኛ 6. ገበሬ 2. ጡረተኛ 7. ስራየሌለው 3. የቤትእመቤት 8. ሌላይገለጽ---- 4. የቀንሰራተኛ 5. ነጋዴ |  |
| 05. | የመኖሪያአድራሻ? | 1. ከተማ 2. ገጠር |  |
| 06. | አማካይወርሃዊየቤተሰብገቢ? | --------------------የኢትዮዽያብር |  |

| **ክፍልሁለት:- ከስነባህሪጋርየተያየዙጥያቄዎች** | | | |
| --- | --- | --- | --- |
| **ተ.ቁ** | **ጥያቄዎች** | **ምላሽ** | **ምርመራ** |
| 01. | ሲጋራአጭሰዉያዉቃሉ? | 1. አዎ 2. የለም | የለምከሆነወደጥያቄ 05 ይለፉ |
| 02. | ለምንያክልጊዜአጭሰዉያዉቃሉ? | 1. ከ 1 አመትበታች 2. ከ 1-5 አመት 3. ከ 5 አመትበላይ |  |
| 03. | በየቀኑያጨሻሉ? | 1. አዎ 2. የለም |  |
| 04. | በቀንምንያክልሲጋራያጨሻሉ? | ---------በቁጥር |  |
| 05. | በዚህ 3 ወርዉስጥየአልኮልመጠጦችንጠጥተዉያዉቃሉ? | 1. አዎን 2. የለም | መልሱየለምከሆነወደጥያቄ 09 ይለፉ |
| 06. | ምንአይነትየአልኮልመጠጦችንተጠቅመዉያዉቃሉ? | 1. ቢራ 3. ጠላ 2. ወይን 4. አረቂ   5.ሌላይገለጽ------- |  |
| 07. | በቀንዉስጥምንያክልየአልኮልመጠጥተጠቅመዉያዉቃሉ? | -------ጠርሙስ/ብርጭቆ/ብርሌ/ጣሳ  ባለ---------ሚሊሊትር |  |
| 08. | በሳምንትዉስጥስንትቀንይጠጣሉ? | ----- ቀን |  |
| 09. | በዚህ 6 ወርዉስጥየሚሰሩትስራብዙጉልበትናሀይልየሚጠይቅየልብምትንናየአተነፋፈስንፍጥነትየሚጨምርነውን? ማለትምመሸከም፣መቆፈር፣ወይምግንባታንወዘተ | 1. አዎ 2. የለም | መልሱየለምከሆነወደጥያቄ 12 ይለፉ |
| 10. | በሳምንትውስጥምንያህልቀናትጉልበትናኃይልየሚጠይቅስራይሰራሉ? | _____ቀናት |  |
| 11. | በቀንውስጥምንያህልደቂቃ/ሰዓትከባድጉልበትናሃይልየሚጠይቅስራእየሰሩያሳልፋሉ? | ------- በሰዓት/በደቂቃ |  |
| 12. | ባለፈዉ 6 ወርዉሰጥወደተለያዩስፍራዎችለመድረስ/ለመንቀሳቀስበእግርወይንም በ ሳይክልጉዞአድርገዉያዉቃሉ? | 1. አዎ 2. የለም |  |
| 13. | በሳምንትውስጥምንያህልቀናትወደተለያዩስፍራዎችለመድረስወይንምለመንቀሳቀስበእግርወይንም በ ሳይክልጉዞአድርገዉያዉቃሉ? | --------ቀናት |  |
| 14. | በቀንውስጥምንያህልሰዓት/ደቂቃበእርምጃወይንም በ ሳይክልከቦታቦታበመዘዋወርያሳልፋሉ | ----በሰዓት/በደቂቃ |  |

| **ክፍልሶስት:-ከስኳርእናከአይንምርመራጋርየተያያዙጥያቄዎች** | | | |
| --- | --- | --- | --- |
| **ተ.ቁ** | **ጥያቄዎች** | **ምላሽ** | **ምርመራ** |
| 01. | ስኳሩንካወቁምንያክልጊዜሆነው? | __________ዓመት |  |
| 02. | ከ ቤተሰብስኳርያለበትአለ? | 1. አዎ 2. የለም |  |
| 03. | ለስኳርህመምክትትልወደሆስፒታሉበየስንትግዜውይመጣሉ? | 1. በየውሩ  2. በየ 3 ወሩ  3. በየ 6 ወር  4. በየአመቱ  5. ሌላካለይጠቀስ--- |  |
| 04. | ከዚህበፊትዓይንዎትንተመርምረውያውቃሉ? | 1. አዎ 2. የለም |  |
| 05. | በስኳርህመምምክንያትየሚመጣየአይንየሁዋለኛዉየብርሐንመቀበያ(ረቲና) ጉዳት/እክልሲባልሰምተዉያዉቃሉ? | 1. አዎ 2. የለም |  |
